# A simulation study comparing aberration detection algorithms for syndromic surveillance

**DOI:** 10.1186/1472-6947-7-6

**Published:** 2007-03-01

**Authors:** Michael L Jackson, Atar Baer, Ian Painter, Jeff Duchin

**Affiliations:** 1 Public Health – Seattle and King County, 999 Third Avenue, Suite 500, Seattle WA, 98104, USA; 2 Department of Epidemiology, University of Washington, Mail Box 357236, Seattle WA, 98195-7236, USA; 3 Foundation for Healthcare Quality, 705 Second Avenue, Suite 703, Seattle WA, 98104, USA

## Abstract

**Background:**

The usefulness of syndromic surveillance for early outbreak detection depends in part on effective statistical aberration detection. However, few published studies have compared different detection algorithms on identical data. In the largest simulation study conducted to date, we compared the performance of six aberration detection algorithms on simulated outbreaks superimposed on authentic syndromic surveillance data.

**Methods:**

We compared three control-chart-based statistics, two exponential weighted moving averages, and a generalized linear model. We simulated 310 unique outbreak signals, and added these to actual daily counts of four syndromes monitored by Public Health – Seattle and King County's syndromic surveillance system. We compared the sensitivity of the six algorithms at detecting these simulated outbreaks at a fixed alert rate of 0.01.

**Results:**

Stratified by baseline or by outbreak distribution, duration, or size, the generalized linear model was more sensitive than the other algorithms and detected 54% (95% CI = 52%–56%) of the simulated epidemics when run at an alert rate of 0.01. However, all of the algorithms had poor sensitivity, particularly for outbreaks that did not begin with a surge of cases.

**Conclusion:**

When tested on county-level data aggregated across age groups, these algorithms often did not perform well in detecting signals other than large, rapid increases in case counts relative to baseline levels.

## Background

In the short time since syndromic surveillance[1] was introduced as an early warning system for detecting outbreaks, considerable effort and expense have gone into developing syndromic surveillance systems. Although there have been substantial developments in the methods and tools used for this practice, the public health value of the various approaches to syndromic surveillance has rarely been evaluated. In particular, a critical component needing further study is the relative accuracy and timeliness of the aberration detection methods of these systems.

In aberration detection, statistical models determine whether the counts in a given syndrome and day are unusually high and thus worth investigating. Many statistical algorithms are available, including control-chart-based models[2,3], scan statistics[4,5], autoregressive moving averages[6], and regression models[7,8]. To optimize outbreak detection, surveillance system designers and users need to understand which methods perform well or poorly in different settings.

Many studies have described the performance of individual aberration detection methods [3-10]. However, multiple algorithms have seldom been compared on the same data [11-14], which is problematic because algorithms that work well for one data source may not do as well for another. Further, studies comparing different algorithms have either tested the algorithms on only a single type of outbreak[11,14], or have used simulated baseline data rather than syndrome counts from real systems[12,13]. Using real system data is important, because it is unknown how well simulated data approximate the relevant features of real syndrome counts. Testing performance on outbreaks of different temporal distributions and sizes is important because of the uncertainty about the types of outbreaks likely to be encountered in practice. To date, no published studies have systematically compared detection methods using real syndromic surveillance data. This lack of comparisons on actual syndromic surveillance data makes it difficult to select aberration detection methods objectively.

To address these limitations, we compared the utility of six commonly-used aberration detection algorithms using data from our syndromic surveillance system at Public Health – Seattle & King County. We simulated syndrome counts that might result from a variety of outbreaks, and added these to actual daily counts from syndromes monitored by our system. We then evaluated the performance of the six algorithms on the resulting data.

## Methods

Our syndromic surveillance system receives data from 18 of 19 emergency departments (EDs) in King County. Each morning, EDs send data on all visits that occurred the previous day, including the date and time of the visit; the patient's age, sex, and home zip code; a free-text chief complaint; diagnosis, if available; and disposition. Chief complaints are classified into syndromes based on the presence or absence of key words using a modified version of the chief complaint coder developed by the New York City Department of Health and Mental Hygiene[15]. For each syndrome, daily case counts are determined both by age group and aggregated across age groups. In this study, we used the aggregated counts. Because historical data were not available from all EDs when we began this study, we restricted our analysis to the nine EDs with complete reporting from 2001–2004.

Because we could not use all of the syndromes we monitor in this analysis, we chose a representative set by grouping syndromes into four categories based on their mean daily counts, and then selecting one syndrome from each group. The final syndromes we used as baselines had mean (standard deviation) daily counts of 60 (16.0), 35 (9.9), 10 (4.0), and 2 (1.6) visits per day. These counts corresponded to ED visits for respiratory illness, influenza-like illness, and asthma syndromes, and pneumonia hospitalizations, respectively. From these four baselines we used data from 2001 through 2003 to provide background counts for the algorithms, and used data from 2004 for testing the algorithms. For each of these syndromes, we calculated the variability in daily counts by weekday and month using Poisson regression [see Additional file 1]. Notably, all four baselines had significant month effects, indicating the presence of seasonal trends. The four baselines also showed significant day-of-week effects.

We compared the performance of six aberration detection methods. We evaluated the three control-chart-based algorithms commonly referred to as C1, C2, and C3[13]. For C1 and C2, the test statistic on day t was calculated as

S_t _= max(0, (X_t _- (μ_t _+ k*σ_t_))/σ_t_)

where X_t _is the count on day t, k is the shift from the mean to be detected, and μ_t _and σ_t _are the mean and standard deviation of the counts during the baseline period. For C1, the baseline period is (t-7,...,t-1); for C2 the baseline is (t-9,...,t-3). The test statistic for C3 is the sum of S_t _+ S_t-1 _+ S_t-2 _from the C2 algorithm.

We evaluated a generalized linear model (GLM), using a three-year baseline and Poisson errors, with terms for day of the week, month, linear time trend, and holidays. The full model for the expected count on day t was

E(X_t_) = β0 + β1(Sunday) + ... + β6(Friday) + β7(January) + ... + β17(November) + β18(Holiday) + β19(time trend)

and the test statistic was the probability from a Poisson distribution of observing at least X_t _cases given E(X_t_).

We also included two Exponential Weighted Moving Average (EWMA) models[16], using a 28-day baseline and smoothing constants of 0.4 (EWMA4) and 0.9 (EWMA9). The smoothed daily counts were calculated as

Y_1 _= X_1_; Y_t _= ω*X_t _+ (1 - ω)*Y_t_-1

and the test statistic was calculated as

T_t _= (Y_t _- μ_t_)/[σ_t_*(ω/(2 - ω))^1/2^]

where ω is the smoothing constant and other notation as defined above. The baseline period for μ_t _and σ_t _was set to (t-30,...,t-3).

### Outbreak simulation

**Figure 1:**
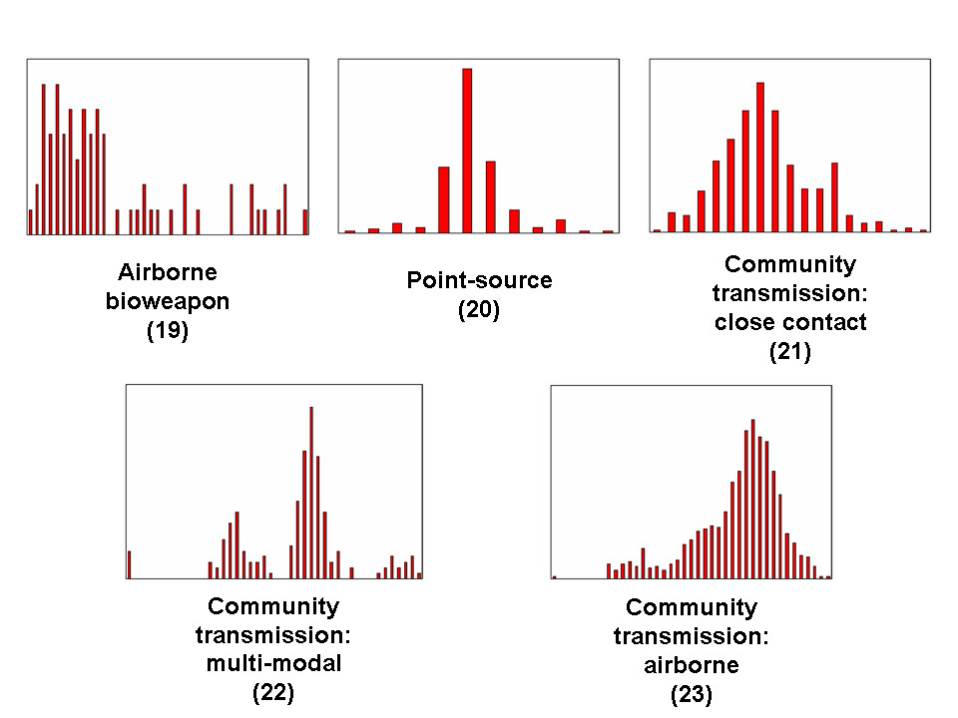
Temporal distributions used for simulating outbreaks, from the epidemic curves of historic outbreaks, with references.

Rather than try to model the full outbreak process from infection to ED visit, as has been done for some limited outbreak types[17], we tried to model a wide range of outbreak signals, representing various ways that outbreaks in the community could alter ED syndrome counts. We based our simulations on the approach described by Mandl et al[18]. First, we chose five temporal distributions, using the epidemic curves of historical outbreaks, to represent several ways in which a pathogen could spread through a community (Figure 1). These were (a) the airborne release of a bioweapon[19]; (b) point-source exposure to an infectious agent[20]; (c) transmission of a pathogen spread by close contact;[21] (d) transmission of an airborne pathogen[22]; and (e) transmission resulting in a multi-modal distribution[23]. Next, we simulated a range of outbreak signal durations (lasting 1, 2, 4, 6, 8, 16, and 32 days) and a range of sizes using the forecast errors of the six algorithms[18]. We determined the forecast errors for each algorithm for each day of 2004, and calculated the standard deviations of these errors. We set the size of the largest outbreak signal to be roughly four times the largest standard deviation, rounded to a convenient number. The largest standard deviation was 13.2 counts, and our outbreak signals ranged from 5 to 50 cases in increments of 5.

Next, we simulated daily outbreak signal counts by creating every unique combination of the temporal distributions, durations, and sizes. Since all outbreaks of one day duration have the same temporal distribution, this gave us 310 different outbreak signals. For each signal, we converted the epidemic curve of the appropriate historical outbreak into an empirical probability distribution. We divided this distribution into the appropriate number of days for the simulated outbreak. We then calculated the cumulative probability of a case occurring on or before day d for each day (1,...,d) of the outbreak signal. Finally, we assigned each case a random number between 0 and 1, chosen from a uniform random distribution. The total number of cases on day d was the sum of all cases whose random number was greater than the cumulative probability for day d-1, and less than or equal to the cumulative probability for day d. This random assignment was repeated for each of the 310 outbreak signals.

### Algorithm testing

We created evaluation datasets by adding the simulated outbreak signals to each of the four baselines, with the outbreak counts starting on January 2nd, 2004. To avoid bias due to day-of-the-week effects and seasonality, we repeated this process starting the outbreak on every other day of 2004 for each of the four baselines. This gave us 183 datasets per outbreak per baseline, for a total of 226,920 datasets for analysis.

We applied the six algorithms to the evaluation datasets, and calculated two outcome measures for each algorithm on each dataset. The first was whether the algorithm ever detected the outbreak signal. The second was the earliest day of an alert, among signals that were detected. The earliest day of alert was counted from the day on which the first simulated case was added. Due to the stochastic nature of these simulations, this was not always the first day of the epidemic. For example, consider a simulated outbreak signal of five cases 32 days duration started on January 2^nd^, 2004. The cases might appear on days 3, 5, 8, 17, and 30 of the signal. In this situation, we would begin counting the days until detection starting from day 3 (January 4^th^).

We calculated these outcomes while running the algorithms at an alert rate of 0.01 (an average of one alert every hundred days). We set this alert rate empirically by applying each algorithm to each baseline without any added outbreak signals, and determining the threshold that would yield an average of one alert per 100 days. Note that in this study we defined an alert rate, rather than a false positive rate (which is 1 – specificity). Calculating a false positive rate (i.e. a specificity) requires assuming that the baseline syndromes did not contain any signals from true outbreaks in the community. This is an unreasonable assumption, given the known yearly influenza epidemics and the probable presence of other unknown outbreaks. By using an alert rate instead of a false positive rate, we allowed for the existence of these signals in our baseline data.

### Comparing algorithm performance

For each of the 310 outbreak signals, in each of the four baseline syndromes, we computed the sensitivity (that is, the probability of detecting the signal given the presence of an outbreak) by averaging the detection outcome across all 183 analysis runs. We also computed the median of the earliest day of detection. We used ANOVA to test for significant differences in the algorithms' sensitivities. Separate tests were conducted by baseline and by outbreak distribution, duration, and size. In each stratum of these four grouping variables we tested for significant differences between algorithms in the probability of detection. We also tested for differences within each algorithm across the strata. For all ANOVA comparisons that were significant at the 0.05 level, we compared the performance of all pairs of algorithms by t-test.

**Figure 2:**
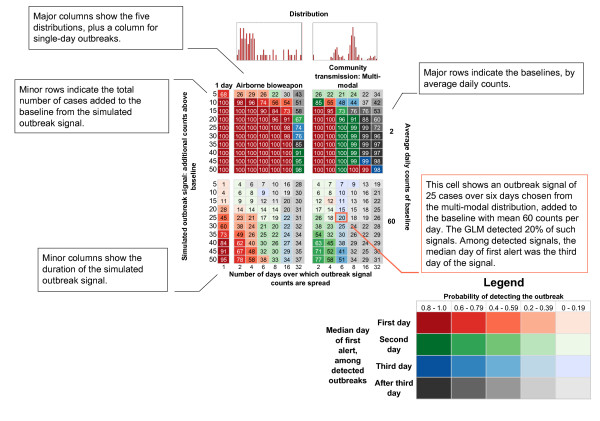
Guide to interpreting Figure 3.

We also present a figure showing the performance of one algorithm on each of the 310 simulated outbreak signals, at each of the four baselines. Because of the multiple comparisons problem, we did not test for significant differences between the cells of the figure. However, we present this figure for qualitative comparisons, so that the reader can visualize the effects of baseline and outbreak temporal distribution, size, and duration on relative algorithm performance, over the range of outbreak signals we tested (Figure 2). All simulations and analyses were performed using SAS version 8.2 (SAS Institute Inc., Cary North Carolina).

## Results

Averaged across all baselines and outbreak signals, C1 detected 34% of outbreaks (95% CI 32–35%); C2 detected 39% (95% CI = 37%–41%); C3 detected 36% (95% CI = 34%–38%); EWMA4 detected 41% (95% CI = 39%–43%); EWMA9 detected 45% (95% CI = 43%–47%); and the GLM detected 54%(95% CI = 52%–56%). In general, the probability of detecting an outbreak was inversely related to average baseline counts (Table 1). Depending on the algorithm, ability to detect an outbreak ranged from 11–28% for the syndrome with mean 60 visits per day, to as high as 62–85% for a syndrome with mean 2 visits per day. Within each baseline, there were significant differences in sensitivity between the algorithms. Overall, when stratified by baseline, the GLM was more sensitive than the other algorithms (p-value < 0.0001 for all pairwise comparisons, except compared to the EWMA methods in the pneumonia hospitalization syndrome, where p <0.05). The GLM's sensitivity was highest (85.3%, 95% CI = 82.5%–88.0%) when the baseline counts were lowest (mean 2 visits per day), and lowest (27.8%, 95% CI = 25.7%–29.8%) when the baseline counts were highest (mean 60 visits per day). The relative performance of C1, C2, C3, EWMA4, and EWMA9 varied across the four baselines.

Of the algorithms evaluated, only the performance of C1 varied across outbreak distributions (p = 0.002) (Table 2). C1 had better detection for the point-source distribution (mean probability of detection 38.1%) compared with the three community-transmission distributions (p < 0.05 for all pairwise t-tests). Although the other algorithms had similar patterns of detection, the differences were not significant at the 0.05 level. Within each outbreak distribution, the GLM had better detection compared with the other algorithms (p < 0.0001 comparing GLM to C1, C2, or C3; p < 0.01 comparing the GLM to EWMA4 and EWMA9), with sensitivities as high as 56.5% for both the airborne bioweapon and the point-source distributions. The sensitivities of the two EWMA models did not differ across distributions (p > 0.05 for all comparison).

Across all six algorithms, the probability of detection increased as the size of the outbreak increased (Table 3). Within each outbreak size, GLM had better detection than the other algorithms (p < 0.001 comparing GLM to C1, C2, or C3; p < 0.05 comparing GLM to EWMA4 or EWMA9). The probability of detection tended to be low for all six algorithms even at the largest outbreak sizes (50 extra cases above the baseline), ranging from 52.9% for C1 (95% CI = 47.0%–58.7%) to 77.4% with GLM (95% CI = 72.9%–81.9%).

Outbreak duration was inversely related to sensitivity for all algorithms (Table 4). The probability of detection was highest for one- and two-day surges in cases. However, even when all excess cases occurred on a single day, detection was fairly poor for most algorithms, ranging as low as 44.5% with C3 (95% CI = 31.0%–58.1%). The GLM was better than all other algorithms at detecting single-day surges in cases (73.4%, 95% CI = 62.4%–84.4%), with the exception of EWMA9, where the difference between the two methods was not significant (p < 0.05). Within each of the other categories of outbreak duration, the GLM had better detection than the other algorithms (p < 0.001 comparing GLM to C1, C2, or C3; p < 0.05 comparing GLM to EWMA4 or EWMA9).

**Figure 3:**
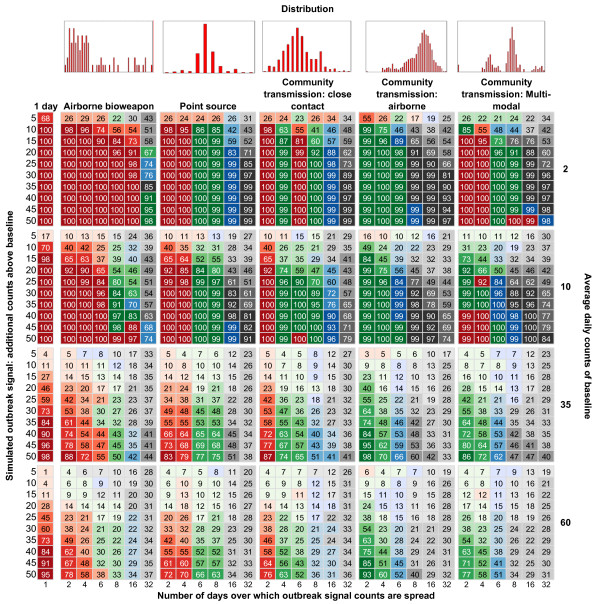
Percent of outbreak signals detected, and median timeliness of detection, for the generalized linear model running at an alert rate of 0.01, for each of 310 outbreak signals on each of four baseline syndromes.

Because the GLM generally detected more outbreaks than the other algorithms when stratified by outbreak size, duration, distribution, or baseline, we present a more detailed view of this algorithm stratified by these four factors simultaneously (Figure 3). As described in Figure 2, each cell in this figure shows the probability of detecting an outbreak for different outbreak signals. To allow for quick qualitative comparisons across outbreak characteristics, the cells are color-coded and shaded, with colors indicating the median day of the first alert among detected outbreaks, and darker shades indicating a higher likelihood of detection.

This stratification suggests several qualitative trends. First, regardless of distribution, the GLM tended to have low sensitivity when the baseline counts for a given syndrome were 35 per day or higher. As shown in Figure 3, outbreak detection under these circumstances was generally below 80%, except in some situations where the outbreak size was large relative to the baseline (i.e., 35–40 excess counts per day) and the surge in cases occurred on a single day. For example, a single-day surge of influenza-like illness resulting in 35 cases above the average daily baseline count of 35 cases was detected in 84% of the tests. That is, a doubling of the average daily case counts on a single day could be reliably detected 84% of the time. This detection level dropped to 55–74% (depending on the distribution) if the excess counts were spread over a period of two days. Sensitivity was poorer with smaller outbreak sizes. For example, a single-day surge of 20 cases of influenza-like illness (a 57% increase over the mean baseline count of 35 cases per day) was detected in less than half (46%) of the tests.

Second, the GLM's performance was generally better for situations where the average daily baseline count was lower (i.e., 10 or fewer cases). In this scenario, the algorithm tended to detect the surge in cases within the first or second day with high probability (>80%). The probability of detection was directly related to outbreak size and inversely related to outbreak duration.

Third, there were some qualitative differences in detection according to outbreak distribution. Distributions where the bulk of the cases occurred early (such as the airborne bioweapon) tended to be detected earlier than slowly-building outbreaks (such as the multi-modal community transmission).

## Discussion

Although public health departments have been quick to adopt syndromic surveillance systems for outbreak detection, few studies have demonstrated system effectiveness. The practical utility of syndromic surveillance depends on several factors, including the quality and accuracy of the source data; the sensitivity, specificity, predictive value, and timeliness of the aberration detection methods; and the user's response to alerts given by the system. In this study, we compared the performance of six aberration detection methods under a broad set of circumstances by adding simulated outbreak signals to actual daily syndrome counts. To our knowledge, this is the largest simulation study comparing syndromic surveillance algorithms that has been published to date. Our evaluation was based on 310 unique outbreak signals, each of which was tested on 183 separate dates in each of four baselines.

Consistent with previous evaluations of syndromic surveillance algorithms[10,12,24], we found that the ability to detect outbreaks was better with larger outbreaks and lower baseline counts. We also observed that detection of a signal of a fixed size increases as the baseline counts decrease, which has been observed elsewhere[13]. This is unsurprising, as a fixed signal size causes a greater relative increase in the case count for a baseline with few daily cases compared to a baseline with many daily cases. Furthermore, our findings suggest that the temporal distribution of cases (i.e., the shape of the epidemic curve) generally does not affect sensitivity but may affect timeliness, which Wang et al. observed in evaluating an autoregressive periodic model (APM)[10].

Beyond these general observations, we had two more specific findings. First, across different baselines, and different characteristics of the outbreak signals, the generalized linear model detected more outbreak signals than the other five algorithms. This was surprising, as we had expected to observe more heterogeneity in the relative performance of the algorithms, particularly across outbreak distributions. Buckeridge et al[25] have suggested that EWMA9 (which approximates a Shewhart chart) should outperform EWMA4 on single-day spikes and scattered signals (such as the multi-modal community transmission), while EWMA4 should be better on lognormal curves that increase rapidly (such as the close-contact community transmission). Furthermore, the control-chart-based methods have been reported to have good sensitivity for detection in rare syndromes, with C3 better suited than C1 and C2 for detecting slowly building outbreaks[13]. Of note, our baseline syndromes all had strong day-of-week trends [see Appendix]; since the GLM included weekday parameters, this may have contributed to its superior performance relative to the other methods, which do not correct for such trends[7].

Secondly, although we found that the GLM tended to perform better than the other methods, all six algorithms performed poorly in many outbreak scenarios. The algorithms were generally able to detect large one- or two-day surges in case counts (where "large" means exceeding twice or three times the standard deviation of the baseline), or signals of longer duration that were very large relative to the baseline. However, these are the types of signals most likely to be detected by the astute clinician (in the case of outpatient or ED data) or by an epidemiologist visually looking for jumps in counts or proportions in a time series. Aberration detection is most needed for detecting low-to-moderate increases in cases and for slowly increasing outbreaks. Yet when run at a rate of one alert per 100 days, none of the algorithms we tested detected these types of signals reliably, suggesting that users run a high risk of missing outbreaks of interest across a wide range of scenarios.

One feature of Figure 3 deserves mention here. As the duration of the signal increases, sensitivity appears to follow either a U-shaped trend (when sensitivity is high in the early days of the outbreak) or to increase with increasing duration. At first glance, this is counter-intuitive, as the ability to detect a signal of a given size should decrease as the cases are distributed over more days. In this case, the superior sensitivity for longer outbreaks does not represent a real benefit, but rather reflects the fact that there is a set alert rate of 0.01. An alert unrelated to the presence of a simulated outbreak, due to variation in the baseline syndrome counts, will occur roughly once every hundred days. It is more likely that such an alert will happen by chance during a simulated signal when that signal occurs over the course of 32 days than when the signal occurs over the course of one or two days.

There are several factors which may limit the generalizability of our findings. First, the results of this study are modelled on the allocation of cases along epidemic curves of a fixed distribution, duration, and size. We did not model the full progression from exposure to disease to healthcare use, as has been done previously for some limited outbreak types[17]. Rather, we attempted to cover a wide array of the outbreak signals that could occur in practice. Thus, our results may not be generalizable to all outbreak settings, because the scenarios we produced may not be entirely representative of the scenarios for which the algorithms were designed to operate[26]. In addition, we set the alert rates for each algorithm empirically, by applying the algorithms to the baseline syndromes and finding the threshold that would yield approximately one alert every 100 days. The sensitivity of the algorithms used in this analysis will likely differ if those same algorithms are applied in systems that use different thresholds. Furthermore, the thresholds that gave an alert rate of 0.01 in our data may yield different alert rates in other data, and may also differ when applied to stratifications of the data, such as by age categories or geographic groupings.

It is a limitation of the syndromic surveillance literature that each evaluation has used different baseline data. The baselines likely differ in terms of random and systematic variation. Furthermore, published studies have rarely reported detailed information about their baseline time series; often, studies have not even reported mean counts or variances. This limitation makes comparisons between studies difficult and extrapolation to other datasets uncertain. We included the appendix, with its detail about the syndromes we used, primarily so that our baselines can be compared with other time series, at least in terms of mean counts, day-of-week effects, and seasonal trends. This gives other users a better basis for comparing our baseline counts with their own syndromic data. However, there may be other features of syndromic time series that affect algorithm performance. The field of syndromic surveillance would greatly benefit from an analysis of the features of syndromic time series that impact detection, and the relative importance of these features.

The poor overall performance of the algorithms we examined raises another question: Are there other algorithms that may perform better? Because this study was computationally intensive, we did not evaluate other algorithms that have been proposed for syndromic surveillance, such as autoregressive models[6,24] or the Pulsar method[11]. We were also unable to evaluate scan statistics[4,5] or other methods that use spatial data, because our simulation method aggregated cases to the county level. Comparing our results to prior studies is difficult, not only because of the different baselines as mentioned above, but because studies have varied in the alert rates at which they have tested the algorithms. Prior studies have tended to use rates between 0.05 (one alert every 20 days) and 0.03 (roughly one alert per month). We feel that these rates are too high for routine surveillance and could desensitize users, leading to a reduced likelihood of following up on any given alert[27]. Because of the problems in comparing evaluations of algorithms across data sets, it is difficult to determine whether other algorithms might have performed better on our data. This remains an area for active research.

## Conclusion

The results of this study suggest that commonly-used aberration detection methods for syndromic surveillance often do not perform well in detecting signals other than large, rapid increases in case counts relative to baseline levels. To the degree that our results are generalizable to other settings, this poor performance may be a feature of other systems as well. These results suggest that users should exercise some caution in reviewing algorithm output. Although the GLM method tended to have better sensitivity overall, there was variability in algorithm performance across outbreak feature sets, illustrating that a one-size-fits-all method is unlikely. Additional work is still needed to develop and evaluate syndromic surveillance algorithms across outbreak signals and to determine the value of these systems in public health practice.

## Competing interests

The author(s) declare that they have no competing interests.

## Authors' contributions

MLJ designed the study, programmed the simulations and analyses, and drafted the manuscript. AB helped conceive the study, was responsible for the data collection, helped design and coordinate the study, and helped draft the manuscript. IP participated in the design of the study and in programming the analyses. JD helped conceive and design the study and draft the manuscript. All authors have read and approved the final manuscript.

## Pre-publication history

The pre-publication history for this paper can be accessed here:



## Supplementary Material

Additional file 1 Appendix. This appendix contains results of Poisson regression analyses on the daily visit counts from the four syndrome time series used in this study. Click here for file

## Figures and Tables

**Table 1:** Mean percent of outbreak signals detected by six aberration detection algorithms, tested on four baseline syndromes, at an alert rate of 0.01

**Syndrome:**		**C1**	**C2**	**C3**	**GLM**	**EWMA 0.4**	**EWMA 0.9**	**F value**	**p-value**
**Pneumonia hospitalizations (mean 2 visits per day)**	Mean	62.0%	69.3%	72.0%	85.3%	80.5%	79.6%	24.94	<0.0001
	Lower CI*	58.3%	65.8%	68.6%	82.5%	77.2%	76.3%		
	Upper CI	65.8%	72.9%	75.5%	88.0%	83.9%	82.9%		
**Asthma visits (mean 10 visits per day)**	Mean	35.4%	52.2%	48.8%	70.0%	52.3%	57.7%	36.63	<0.0001
	Lower CI	31.7%	48.5%	45.1%	66.5%	48.6%	53.9%		
	Upper CI	39.0%	56.0%	52.5%	73.4%	56.0%	61.5%		
**Influenza-like illness visits (mean 35 visits per day)**	Mean	21.2%	17.9%	11.1%	34.4%	19.0%	28.2%	84.08	<0.0001
	Lower CI	19.6%	16.5%	10.4%	31.9%	17.4%	26.0%		
	Upper CI	22.9%	19.2%	11.9%	36.9%	20.5%	30.4%		
**Respiratory visits (mean 60 visits per day)**	Mean	16.4%	17.1%	10.8%	27.8%	13.3%	15.7%	84.90	<0.0001
	Lower CI	15.1%	16.0%	10.3%	25.7%	12.5%	14.5%		
	Upper CI	17.6%	18.3%	11.4%	29.8%	14.0%	16.9%		
**F value**		205.58	349.50	531.70	398.73	550.30	406.76		
**p-value**		<0.0001	<0.0001	<0.0001	<0.0001	<0.0001	<0.0001		

* CI = 95% confidence interval

**Table 2:** Mean percent of outbreak signals detected by six aberration detection algorithms, tested on five outbreak distributions, at an alert rate of 0.01

**Distribution:**		**C1**	**C2**	**C3**	**GLM**	**EWMA 0.4**	**EWMA 0.9**	**F value**	**p-value**
**Airborne bioweapon**	Mean	37.4%	41.5%	36.5%	56.5%	41.6%	46.9%	12.85	<0.0001
	Lower CI*	33.5%	37.5%	32.4%	52.4%	37.5%	42.7%		
	Upper CI	41.2%	45.4%	40.7%	60.5%	45.8%	51.1%		
**Point-source**	Mean	38.1%	41.7%	38.0%	56.5%	42.4%	47.5%	9.49	<0.0001
	Lower CI	33.9%	37.3%	33.5%	52.1%	37.7%	42.8%		
	Upper CI	42.3%	46.2%	42.6%	60.9%	47.0%	52.1%		
**Community transmission: close contact**	Mean	32.2%	37.2%	34.6%	53.0%	40.1%	43.7%	12.11	<0.0001
	Lower CI	28.3%	33.1%	30.2%	48.7%	35.6%	39.3%		
	Upper CI	36.0%	41.4%	38.9%	57.3%	44.6%	48.1%		
**Community transmission: airborne**	Mean	32.1%	37.5%	34.8%	52.8%	41.2%	44.1%	11.57	<0.0001
	Lower CI	28.3%	33.4%	30.4%	48.4%	36.7%	39.6%		
	Upper CI	36.0%	41.7%	39.2%	57.2%	45.8%	48.6%		
**Community transmission: multi-modal**	Mean	28.4%	37.4%	34.4%	52.6%	40.9%	43.9%	15.05	<0.0001
	Lower CI	25.1%	33.2%	30.1%	48.2%	36.3%	39.4%		
	Upper CI	31.7%	41.5%	38.8%	57.0%	45.5%	48.4%		
**F value**		4.39	1.22	0.05	0.86	0.13	0.65		
**p-value**		0.0016	0.30	0.74	0.4881	0.97	0.63		

* CI = 95% confidence interval

**Table 3:** Mean percent of outbreak signals detected by six aberration detection algorithms, tested on six outbreak sizes, at an alert rate of 0.01

**Size:**		**C1**	**C2**	**C3**	**GLM**	**EWMA 0.4**	**EWMA 0.9**	**F value**	**p-value**
**5 cases**	Mean	10.4%	11.9%	9.6%	16.0%	10.3%	12.0%	9.55	<0.0001
	Lower CI*	8.9%	10.5%	8.3%	14.1%	9.0%	10.6%		
	Upper CI	11.8%	13.3%	10.9%	18.0%	11.7%	13.5%		
**10 cases**	Mean	16.4%	18.8%	15.4%	30.2%	20.2%	22.9%	10.01	<0.0001
	Lower CI	13.7%	16.2%	13.0%	25.7%	16.7%	19.0%		
	Upper CI	19.2%	21.5%	17.9%	34.6%	23.8%	26.7%		
**20 cases**	Mean	26.7%	32.2%	29.4%	48.4%	34.8%	38.4%	8.17	<0.0001
	Lower CI	22.1%	27.3%	24.3%	42.5%	29.0%	32.7%		
	Upper CI	31.3%	37.2%	34.5%	54.3%	40.6%	44.2%		
**30 cases**	Mean	38.9%	46.2%	42.5%	63.0%	47.8%	52.5%	7.42	<0.0001
	Lower CI	33.3%	40.0%	36.0%	57.2%	41.3%	46.1%		
	Upper CI	44.5%	52.4%	49.0%	68.7%	54.3%	58.9%		
**40 cases**	Mean	47.1%	53.7%	50.0%	71.6%	56.4%	61.6%	8.11	<0.0001
	Lower CI	41.2%	47.4%	43.2%	66.6%	49.8%	55.4%		
	Upper CI	53.0%	59.9%	56.9%	76.6%	63.0%	67.7%		
**50 cases**	Mean	52.9%	58.8%	53.8%	77.4%	62.7%	67.9%	9.83	<0.0001
	Lower CI	47.0%	52.8%	47.0%	72.9%	56.5%	62.2%		
	Upper CI	58.7%	64.8%	60.6%	81.9%	68.9%	73.7%		
**F value**		35.81	38.67	31.48	64.53	37.68	46.16		
**p-value**		<0.0001	<0.0001	<0.0001	<0.0001	<0.0001	<0.0001		

* CI = 95% confidence interval

**Table 4:** Mean percent of outbreak signals detected by six aberration detection algorithms, tested on seven outbreak durations, at an alert rate of 0.01

**Duration:**		**C1**	**C2**	**C3**	**GLM**	**EWMA 0.4**	**EWMA 0.9**	**F value**	**p-value**
**1 day**	Mean	59.8%	58.2%	44.5%	73.4%	52.6%	64.6%	2.58	0.03
	Lower CI*	47.5%	45.7%	31.0%	62.4%	39.6%	52.4%		
	Upper CI	72.2%	70.7%	58.1%	84.4%	65.5%	76.8%		
**2 days**	Mean	47.3%	49.1%	42.4%	64.2%	47.9%	55.2%	7.81	<0.0001
	Lower CI	42.0%	43.7%	36.7%	59.2%	42.3%	49.8%		
	Upper CI	52.6%	54.4%	48.0%	69.2%	53.5%	60.7%		
**4 days**	Mean	39.6%	43.5%	40.1%	57.7%	43.6%	48.9%	6.43	<0.0001
	Lower CI	34.5%	38.2%	34.7%	52.6%	38.2%	43.5%		
	Upper CI	44.7%	48.8%	45.5%	62.7%	49.1%	54.4%		
**6 days**	Mean	33.2%	39.8%	37.6%	54.0%	41.7%	45.5%	7.65	<0.0001
	Lower CI	28.5%	34.7%	32.4%	48.9%	36.4%	40.2%		
	Upper CI	37.8%	44.9%	42.9%	59.0%	47.1%	50.8%		
**8 days**	Mean	27.7%	36.4%	34.7%	51.3%	39.2%	42.2%	10.55	<0.0001
	Lower CI	23.8%	31.7%	29.8%	46.3%	34.0%	37.2%		
	Upper CI	31.6%	41.2%	39.7%	56.2%	44.3%	47.3%		
**16 days**	Mean	23.0%	31.0%	30.2%	47.7%	36.5%	38.3%	17.22	<0.0001
	Lower CI	20.5%	27.4%	26.1%	43.2%	32.0%	33.9%		
	Upper CI	25.5%	34.6%	34.4%	52.2%	41.0%	42.7%		
**32 days**	Mean	26.5%	31.2%	27.4%	47.5%	36.5%	37.5%	36.25	<0.0001
	Lower CI	25.5%	29.7%	25.0%	44.2%	33.1%	34.6%		
	Upper CI	27.5%	32.8%	29.8%	50.8%	39.8%	40.3%		
**F value**		35.81	38.67	31.48	64.53	37.68	46.16		
**p-value**		<0.0001	<0.0001	<0.0001	<0.0001	<0.0001	<0.0001		

* CI = 95% confidence interval
