# Supplementary material for: A simulation study comparing aberration detection algorithms for syndromic surveillance
Source: BMC Med Inform Decis Mak. 2007 Mar 1;7:6. doi: 10.1186/1472-6947-7-6 (PMC1821319; doi:10.1186/1472-6947-7-6)
Supplement: Additional file 1 — Appendix. This appendix contains results of Poisson regression analyses on the daily visit counts from the four syndrome time series used in this study. [file 1472-6947-7-6-S1.doc]

Appendix: Characteristics of the four syndromes chosen as baseline time series for this study. Beta coefficients from Poisson regression analysis for the difference in daily counts by weekday and by month.

|  | Pneumonia hospitalizations | | | Asthma | | | Influenza-like illness | | | Respiratory | | |
| --- | --- | --- | --- | --- | --- | --- | --- | --- | --- | --- | --- | --- |
| Parameter | Mean counts | Standard deviation | p-value | Mean counts | Standard deviation | p-value | Mean counts | Standard deviation | p-value | Mean counts | Standard deviation | p-value |
| Intercept (A Sunday in June) | 0.482 | 0.084 | <0.001 | 2.233 | 0.037 | <0.001 | 3.757 | 0.018 | <0.001 | 4.031 | 0.015 | <0.001 |
| Weekday: |  |  |  |  |  |  |  |  |  |  |  |  |
| Monday | 0.140 | 0.067 | 0.036 | -0.004 | 0.029 | 0.879 | -0.279 | 0.015 | <0.001 | -0.014 | 0.012 | 0.226 |
| Tuesday | 0.086 | 0.067 | 0.200 | -0.152 | 0.031 | <0.001 | -0.366 | 0.015 | <0.001 | -0.134 | 0.012 | <0.001 |
| Wednesday | -0.081 | 0.070 | 0.251 | -0.192 | 0.031 | <0.001 | -0.379 | 0.015 | <0.001 | -0.139 | 0.012 | <0.001 |
| Thursday | -0.060 | 0.070 | 0.389 | -0.159 | 0.031 | <0.001 | -0.370 | 0.015 | <0.001 | -0.172 | 0.012 | <0.001 |
| Friday | 0.010 | 0.069 | 0.881 | -0.164 | 0.031 | <0.001 | -0.356 | 0.015 | <0.001 | -0.143 | 0.012 | <0.001 |
| Saturday | 0.015 | 0.069 | 0.823 | -0.130 | 0.030 | <0.001 | -0.124 | 0.014 | <0.001 | -0.088 | 0.012 | <0.001 |
| Month: |  |  |  |  |  |  |  |  |  |  |  |  |
| January | 0.415 | 0.091 | <0.001 | 0.221 | 0.042 | <0.001 | 0.339 | 0.021 | <0.001 | 0.411 | 0.016 | <0.001 |
| February | 0.428 | 0.092 | <0.001 | 0.363 | 0.042 | <0.001 | 0.430 | 0.021 | <0.001 | 0.498 | 0.016 | <0.001 |
| March | 0.409 | 0.091 | <0.001 | 0.372 | 0.041 | <0.001 | 0.456 | 0.020 | <0.001 | 0.460 | 0.016 | <0.001 |
| April | 0.320 | 0.093 | 0.001 | 0.179 | 0.043 | <0.001 | 0.179 | 0.021 | <0.001 | 0.244 | 0.017 | <0.001 |
| May | 0.265 | 0.094 | 0.005 | 0.122 | 0.043 | 0.005 | 0.032 | 0.022 | 0.145 | 0.114 | 0.017 | <0.001 |
| July | 0.040 | 0.099 | 0.686 | -0.274 | 0.048 | <0.001 | -0.061 | 0.023 | 0.007 | -0.137 | 0.019 | <0.001 |
| August | -0.172 | 0.104 | 0.099 | -0.181 | 0.046 | <0.001 | -0.083 | 0.023 | <0.001 | -0.201 | 0.019 | <0.001 |
| September | -0.047 | 0.102 | 0.644 | 0.187 | 0.043 | <0.001 | -0.125 | 0.023 | <0.001 | -0.004 | 0.018 | 0.839 |
| October | 0.042 | 0.099 | 0.668 | 0.218 | 0.042 | <0.001 | -0.081 | 0.023 | <0.001 | 0.184 | 0.017 | <0.001 |
| November | 0.325 | 0.093 | 0.001 | 0.319 | 0.042 | <0.001 | 0.245 | 0.021 | <0.001 | 0.350 | 0.017 | <0.001 |
| December | 0.433 | 0.091 | <0.001 | 0.331 | 0.041 | <0.001 | 0.435 | 0.020 | <0.001 | 0.465 | 0.016 | <0.001 |
| Overall significance tests: |  |  |  |  |  |  |  |  |  |  |  |  |
| Weekday |  |  | 0.0158 |  |  | <0.001 |  |  | <0.001 |  |  | <0.001 |
| Month |  |  | <0.001 |  |  | <0.001 |  |  | <0.001 |  |  | <0.001 |
